# The quality of life impact of the COVID-19 pandemic and lockdowns for people living with multiple sclerosis (MS): evidence from the Australian MS Longitudinal Study

**DOI:** 10.1007/s11136-024-03620-4

**Published:** 2024-04-05

**Authors:** Glen J. Henson, Ingrid van der Mei, Bruce V. Taylor, Paul Blacklow, Suzi B. Claflin, Andrew J. Palmer, Carol Hurst, Julie A. Campbell

**Affiliations:** 1 Menzies Institute for Medical Research, University of Tasmania, 17 Liverpool St, Hobart, 7000 Australia; 2 Tasmanian School of Business and Economics, University of Tasmania, Churchill Avenue, Sandy Bay, 7005 Australia

**Keywords:** Health economics, Quality of life, COVID-19, Multiple sclerosis, Australia

## Abstract

**Purpose:**

People living with multiple sclerosis (PwMS) in metropolitan Victoria, Australia, experienced a 112-day, COVID-19-related lockdown in mid-2020. Contemporaneously, Australian PwMS elsewhere experienced minimal restrictions, resulting in a natural experiment. This study investigated the relationships between lockdowns, COVID-19-related adversity, and health-related quality of life (HRQoL). It also generated health state utilities (HSU) representative of changes in HRQoL.

**Methods:**

Data were extracted from Australian MS Longitudinal Study surveys, which included the Assessment of Quality of Life-Eight Dimensions (AQoL-8D) instrument and a COVID-19 questionnaire. This COVID-19 questionnaire required participants to rank their COVID-19-related adversity across seven health dimensions. Ordered probits were used to identify variables contributing to adversity. Linear and logit regressions were applied to determine the impact of adversity on HRQoL, defined using AQoL-8D HSUs. Qualitative data were examined thematically.

**Results:**

*N* = 1666 PwMS (average age 58.5; 79.8% female; consistent with the clinical presentation of MS) entered the study, with *n* = 367 (22.0%) exposed to the 112-day lockdown. Lockdown exposure and disability severity were strongly associated with higher adversity rankings (*p* < 0.01). Higher adversity rankings were associated with lower HSUs. Participants reporting major adversity, across measured health dimensions, had a mean HSU 0.161 (*p* < 0.01) lower than participants reporting no adversity and were more likely (OR: 2.716, *p* < 0.01) to report a clinically significant HSU reduction. Themes in qualitative data supported quantitative findings.

**Conclusions:**

We found that COVID-19-related adversity reduced the HRQoL of PwMS. Our HSU estimates can be used in health economic models to evaluate lockdown cost-effectiveness for people with complex and chronic (mainly neurological) diseases.

**Supplementary Information:**

The online version contains supplementary material available at 10.1007/s11136-024-03620-4.

## Introduction

### Multiple sclerosis

Multiple sclerosis (MS) is an autoimmune/neurodegenerative disorder that causes central nervous system demyelination and subsequent neuronal loss [1, 2]. Symptoms associated with MS are protean and may include motor, incoordination, cognitive, sexual, bladder and bowel dysfunction, sensory impairment, pain, and fatigue [3]. Consequently, people living with MS will have varying experiences of the disease. The global prevalence of MS increased to 2.8 million people in 2020, compared to 2.3 million in 2013 [4]. Similarly, our group established that MS prevalence in Australia was 33,335 in 2021, and it is increasing at an accelerating rate [5–7].

### Context of the study

An Australian, state-government-mandated COVID-19 lockdown was enacted in the state of Victoria’s metropolitan region and Mitchell Shire (an area containing 5.2 million people) from July 7th to October 27th, 2020 [8]. The lockdown was introduced in response to a second, regional ‘wave’ of COVID-19 cases, and severely restricted civilian movement and business and service operations (with additional restrictions from August 2nd) [8, 9]. From September 13th, restrictions were sequentially eased [10]. Simultaneously, the remainder of Australia experienced limited restrictions, resulting from a lack of substantial COVID-19 incidence. This generated a natural experiment, with the 112-day Victorian lockdown (“the metropolitan lockdown”) as a pseudo-exposure, which was utilised in this study.

The impetus for this study comes from previous work which has suggested that people living with MS may have been particularly affected by the metropolitan lockdown. For example, one study illustrated that lockdown could prevent people living with MS from accessing non-essential health and care services, which could reduce their quality of life [11]. In addition, lockdowns can isolate people with complex and chronic diseases from support networks, increasing the risk of mental health disorders in an already at-risk population [12].

### Review of existing evidence

To our knowledge, no large quantitative studies, relevant to the impact of the COVID-19 pandemic (hereon referred to as “the pandemic”) and lockdowns on Australian people living with MS, exist. Furthermore, no known study has developed and applied a COVID-19-specific instrument to a cohort of people living with MS to ascertain their perceived adversity due to the pandemic and lockdowns. While HRQoL instruments have been used to evaluate the impact of the pandemic and isolation quarantines on people living with MS, no study has used the Assessment of Quality of Life-Eight Dimensions (AQoL-8D) multi-attribute utility instrument (MAUI) [13].

The AQoL-8D contains 35 survey items relating to eight dimensions of health (physical: independent living, pain, senses; psychosocial: happiness, relationships, coping, self-worth, mental health), and two super-dimensions (physical and psychosocial) [13]. As with other MAUIs, the AQoL-8D generates health state utilities (HSUs), which are measures of health-related quality of life [13]. This instrument had been found to effectively capture psychosocial and subjective wellbeing, in addition to conventional HRQoL, thereby providing HSUs that are representative of a robust and holistic conception of HRQoL [13].

### Aims of the study

This study aimed to determine whether Australian people living with MS exposed to COVID-19-related lockdown experienced greater COVID-19-related adversity, and how this adversity may have impacted their AQoL-8D HSUs, and therefore their HRQoL. The HSU values generated by this study were to be applicable in health economic models. We hypothesised (1) that COVID-19-related adversity would be positively associated with exposure to lockdown for people living with MS and (2) that this adversity would be associated with clinically significant reductions in health-related quality of life. We also hypothesised that (3) COVID-19-related adversity mediated the relationship between HSU and lockdown exposure.

## Methods

### Source of study participants

Study participants were sourced from the Australian Multiple Sclerosis Longitudinal Study, a representative and survey-based cohort study conducted by MS Australia since 2001, with an estimated 96% of participants diagnosed with MS under the McDonald criteria [14, 15]. Recruitment to the study is ongoing, with all participants required to provide informed consent. Ethics approval for the Australian Multiple Sclerosis Longitudinal Study was received from the Tasmania Health and Medical Human Research Ethics Committee (ethics approval number H0014183).

### Sources of data

The principal data source for cross-sectional analysis was the Australian Multiple Sclerosis Longitudinal Study 2020 Quality of Life Survey (August 2020–September 2020; abbreviated as 2020QoL), which was disseminated during the metropolitan lockdown. The AQoL-8D MAUI and the specialised COVID-19 questionnaire were included in this comprehensive and randomised survey, which also included other questions and instruments. Overall, the 2020QoL required approximately 30 minutes to complete via phone, mail, or online.

The 2020QoL also collected clinical and sociodemographic data, as well as qualitative free-text data. Free-text data comprised participant responses to the question “Would you like to provide any other information on your physical or emotional circumstances regarding the COVID-19 pandemic?” Select data used in this study from other Australian Multiple Sclerosis Longitudinal Study surveys was linked to the 2020QoL using unique participant identifiers. Additional detail regarding data collection is included in Appendix 1.

Hot-deck imputation was used to resolve missing data in the COVID-19-related adversity variables and multiple imputation by chained equations was applied to rectify missingness in other variables. Appendix 1 provides further detail regarding data imputation and data missingness. Notably, there was minimal data missingness across all variables.

### Exposure measures: COVID-19-related adversity and lockdown

Adversity related to the pandemic and lockdown was measured on six-point Likert scales (see Supplementary Fig. 1). A score of 0 indicated a positive response, 1 a neutral response, and 2–5 increasing levels of adversity. Following analysis, the six-point scales were recategorised into three-point scales (≤ 1 no adversity [0]; = 2 minor adversity [1]; ≥ 3 major adversity [2]). Positive and neutral responses were combined into the no adversity category following trials of positive response indicator variables in study models. These trials found the indicators to be clinically insignificant (and frequently statistically insignificant) in multivariable regressions (see Supplementary Table 1). Scores of 3 and above were aggregated as differences in the HSU decrements associated with these levels were not statistically significant or otherwise not clinically meaningful (data not shown). Results were robust to the recategorisation.

The measures of COVID-19-related adversity were associated with the following dimensions of health: emotional health, self-care activities, coping, carer relationships, familial relationships, living arrangements, and finances. A composite, categorical indicator of COVID-19-related adversity was also defined. This variable comprised a simple average of reorganised adversity scores. The composite adversity variable was simplified so that it could be represented on the three-point Likert scale and is described in Appendix 2.

Lockdown exposure was defined as possessing a principal residence in a local government area where the lockdown was enforced. Therefore, participants reporting postcodes within affected local government areas were considered exposed to lockdown, with other participants considered unexposed. See Appendix 2 for a detailed summary of the measurement of lockdown exposure, how the associated variable was defined, and a sensitivity analysis that tested the assumptions of this definition.

### Outcome measure: health state utility

The net effect of adversity related to the pandemic and lockdowns was determined using HSUs, generated by the AQoL-8D, as a measure of HRQoL. AQoL-8D HSUs represent levels of health on a continuous scale from 0 (anchored to death) to 1 (equivalent to full health) and are unique in that they effectively capture subjective wellbeing and are particularly sensitive to changes in psychosocial dimensions of health [13, 16]. The AQoL-8D minimum important difference (MID; 0.08 utility points—the smallest change in HSU which is deemed to be clinically meaningful) and general population norm (mean HSU 0.80, SD 0.19) were sourced from the literature for use in the interpretation of results [17, 18].

### Sociodemographic and clinical measures

Sociodemographic and clinical covariates used in the study included: age (years), sex (female or male [reference]), employment status (employed or unemployed [reference]), MS phenotype (relapsing or progressive onset [reference]), education level (postgraduate degree, bachelor’s degree, occupation certificate or diploma, secondary education only or less [reference]), socioeconomic area (measured using the socioeconomic index for areas’ index of relative socioeconomic advantage and disadvantage (IRSAD); categorised into quintiles with the lowest quintile as the reference), and disability severity, measured using the Patient Determined Disease Steps (PDDS) and mapped to Expanded Disease Severity Scale (EDSS) categories. Disability severity was mapped as follows: PDDS of 1 = nil (reference), PDDS of 2 or 3 = mild, PDDS of 4 or 5 = moderate, and PDDS of 6 through 8 = severe. Participants reporting a PDDS of 9, indicating substantial and debilitating sensory, rather than ambulatory, symptoms were categorised as having mild disability severity [19].

### Descriptive analysis and qualitative analysis

Descriptive analyses consisted of three elements. First, COVID-19-related adversity variables were stratified over the categories of key covariates and lockdown exposure. This analysis demonstrated whether variables of interest were crudely associated with COVID-19-related adversity. Second, HSU was stratified by the COVID-19-related adversity variables and lockdown exposure. This allowed for investigation into how the pandemic and lockdowns may have directly impacted HRQoL. Lastly, the number of participants reporting the different ranks of COVID-19-related adversity was determined. This indicated how widespread the impact on the COVID-19-pandemic was among people living with multiple sclerosis, which provided context for study results. All quantitative analyses were undertaken using Stata 17 (StataCorp, 2021).

The qualitative analyses provide otherwise unobtainable insights into the lived experiences of people living with MS during the COVID-19 pandemic. This provided deeper contextualisation and nuance to study results [20, 21]. Analysis of qualitative data utilised NVivo software (QSR International, 2020) to identify key themes relating to the lived experiences of people living with MS during the pandemic.

### Regression analyses

Table 1 provides an integrated explanation of how each of the following regression analyses contributed to the study’s aims. Table 1 also provides summaries of model specifications. All regression analyses utilised robust standard errors.

**Table 1:** Purposes of regression analyses (ordered probit, linear, and logistic) and summaries of model specifications

Model	Purpose	General form	Explanatory notes
Ordered probit	Investigate how lockdown exposure contributed to COVID-19-related adversity ^a^. Identify other covariates associated with COVID-19-related adversity ^a^	$A_{{{\text{ij}}}} = \hat{\varvec{\rho }}_{\text{1j}} .{\varvec{C}}_{{\text{i}}} + \hat{\beta }_{\text{1j}} .L_{{\text{i}}} + e_{{1{\text{ij}}}}$	where *A*_ij_ is the COVID-19-related adversity rank for the ith person and the jth COVID-19 adversity variable, ***C***_i_ is a vector of sociodemographic and clinical covariates, *L*_i_ is a lockdown indicator (zero [not exposed], one [exposed]) and *e*_1i_ is model residuals
Linear (Multivariable)	Determine how COVID-19-related adversity impacted health state utility ^b^. Analyse the direct impact of lockdown on health state utility ^c^	${\text{HSU}}_{{\text{i}}} = \alpha_{1} + \hat{\varvec{\rho }}_{2} .{\varvec{C}}_{{\text{i}}} + \hat{\varvec{\beta }}_{2} .{\varvec{A}}_{{\text{i}}} + e_{{2{\text{i}}}}$	where HSU_i_ is health state utility, *α*_1_ is a constant, ***C***_i_ is a vector of sociodemographic and clinical covariates, ***A***_i_ is a vector of COVID-19 adversity variables, and *e*_2i_ is model residuals
Logistic	Establish an association between COVID-19-related adversity and health state utility in the absence of potential confounding by unobserved, time-invariant factors ^b^	$\Delta {\text{HSU}}_{{\text{i}}} = \alpha_{2} + \hat{\beta }_{2} .\Delta {\text{EDSS}}_{{\text{i}}} + \hat{\beta }_{3} .A_{{\text{i}}} + e_{{3{\text{i}}}}$	where ΔHSU_i_ is an indicator for change in health state utility (zero if less than 0.08, one otherwise), *α*_2_ is baseline odds, ΔEDSS_i_ is change is MS-related disability severity, *A*_i_ is a COVID-19 adversity variable, and *e*_3i_ is model residuals

Superscript letters connect model purposes to the hypotheses in Sect. "Aims of the study". We hypothesised that (a) COVID-19-related adversity would be positively associated with exposure to lockdown for people living with MS, (b) this adversity would be associated with clinically significant reductions in health-related quality of life, and (c) COVID-19-related adversity mediated the relationship between HSU and lockdown

Ordered probits were used to determine which variables (including lockdown exposure) were associated with reports of COVID-19-related adversity (Table 1). For these models, coefficients represented sentiment towards reporting higher COVID-19-related adversity rankings. Therefore, higher coefficients indicated a stronger association between study covariates and COVID-19-related adversity. This explanation is supported by Appendix 3, which provides a comprehensive overview of ordered probits.

Univariable regressions demonstrated crude associations between HSU, and lockdown exposure and COVID-19-related adversity. Multivariable linear regressions were primarily used to estimate association between health state utility, and composite adversity or lockdown exposure (Table 1). Respectively, these regressions demonstrated the direct impact of lockdown exposure, and the general impact of COVID-19-related adversity, on HRQoL. All multivariable linear regressions controlled for all clinical and sociodemographic covariates. Multivariable linear regression was also used to determine whether COVID-19-related adversity associated with a particular domain of health (as measured by the COVID-19 survey) had a greater impact on HSU than COVID-19-related adversity affecting other health domains. These linear regression models employed all COVID-19-related adversity variables simultaneously.

Lastly, logistic regressions demonstrated the association between clinically meaningful reductions in HSU and COVID-19-related adversity. A dependent variable was generated for these models comprising the difference in participants’ HSUs before and during the pandemic. This variable was dichotomised into two categories: no clinically meaningful decrease in HSU (< 0.08) and a clinically meaningful decrease in HSU (> 0.08). A binary form was used as it could discern between clinically significant and insignificant reductions in HSU. As the dependent variable represented change in HSU between two time-points, the associations estimated using logistic regression could not be confounded by unobserved, time-invariant factors. Therefore, the logit regressions provided additional evidence regarding the relationship between COVID-19-related adversity and HSU (Table 1).

Following from above, time-invariant covariates were excluded from logistic regression models. However, all logistic regressions controlled for changes in disability severity, as this was variable over time. Note that adversity variables were used individually in logistic regressions due to high multicollinearity affecting variable significance in multivariable regression. Appendix 3 supports the above explanation with additional information.

## Results

### Characteristics of participants and non-participants

Of the 2513 persons invited to complete the 2020QoL survey, 1683 (67.0%) responded (aligning with the response rates of other Australian Multiple Sclerosis Longitudinal Study surveys), with *n* = 1666 (66.3%) providing sufficiently complete responses to key survey questions for inclusion in this study. Due to incomplete data, the number of participants included in descriptive analyses differs from the number included in the study (*n* = 1666). Though data was imputed, multiple imputed data was only applicable in regressions.

Characteristics of participants and non-participants (*n* = 847) are displayed in Table 2. 2020QoL participants were 79.8% (*n* = 1330) female, and their mean (SD) age was 58.5 (11.3) years. This is consistent with the presentation of MS (disproportionately affects women at a 3:1 ratio, with disease onset often occurring in early adulthood) [2, 22]. Of these participants, 22.0% (*n* = 367) were exposed to the metropolitan lockdown, compared to only 17.2% (*n* = 146) of the non-participants. The only other substantial difference between these subgroups was IRSAD ranking, with participants more likely to reside in fifth quintile postcodes (34.4% versus 29.9%%), reflecting higher socioeconomic status.

**Table 2:** Characteristics of participants and non-participants

	Participants	Non-participants
Total participants, *n*. (% of total)	1666 (66.3)	847 (33.7)
Mean age, years (SD)	58.5 (11.3)	56.8 (11.9)
Sex (Female), *n* (% of subgroup)	1330 (79.8)	663 (78.3)
Employment status, *n* (% of subgroup)		
Yes	504 (30.3)	168 (19.8)
No	819 (49.2)	253 (29.9)
Unknown	343 (20.6)	426 (50.3)
MS phenotype, *n* (% of subgroup)		
Progressive onset	230 (13.8)	104 (12.3)
Relapse onset	1296 (77.8)	470 (55.5)
Unknown	139 (8.3)	273 (32.2)
Education, *n* (% of subgroup)		
Secondary or less	422 (25.3)	174 (20.5)
Certificate or diploma	579 (34.8)	197 (23.3)
Bachelor’s degree	359 (21.6)	150 (17.7)
Postgraduate degree	295 (17.7)	109 (12.9)
Unknown	11 (0.7)	217 (25.6)
Disability severity, *n* (% of subgroup)		
Nil	399 (24.0)	153 (18.1)
Mild	340 (20.4)	228 (26.9)
Moderate	602 (36.1)	162 (19.1)
Severe	314 (18.9)	82 (9.7)
Unknown	11 (0.7)	222 (26.2)
Socioeconomic area, *n* (% of subgroup)^a^		
Quintile one	190 (11.4)	100 (11.8)
Quintile two	264 (15.8)	136 (16.0)
Quintile three	301 (18.1)	164 (19.4)
Quintile four	338 (20.3)	189 (22.3)
Quintile five	572 (34.3)	253 (29.9)
Unknown	1 (0.1)	5 (0.6)
Lockdown, *n* (% of subgroup)		
Metropolitan exposure	367 (22.0)	146 (17.2)
Regional exposure	68 (4.1)	N/A ^b^
Unexposed	1231 (73.9)	701 (82.8)

^a ^Higher quintiles indicate greater socioeconomic advantage. ^b^As non-responses are not dated, and the regional lockdown started after the 2020 Quality of Life survey was initially distributed, exposure to the regional lockdown cannot be calculated for non-respondents

### Stratifications of COVID-19-related adversity variables over key sociodemographic and clinical variables

Key covariate stratifications are presented in Table 3. Age was found to be generally negatively associated with perceptions of COVID-19-related adversity. For example, across categories of age mean adversity rankings in the domain of emotional health were 0.99, 0.82, 0.78, 0.66, and 0.44. Conversely, disability severity was positively associated with reported COVID-19-related adversity. However, this relationship did not persist into the severe disability category. For instance, mean adversity rankings in the domain of coping with MS were 0.48, 0.59, 0.73, and 0.59 (from the lowest to highest disability severity category).

**Table 3:** Stratifications of COVID-19-related adversity over age, disability severity, and sex and lockdown categories

Results are presented as: mean (SD)	Age				
	< 45 (*n* = 157)	45–54 (*n* = 443)	55–64 (*n* = 535)	65–74 (*n* = 389)	> 74 (*n* = 142)
Composite adversity	0.65 (0.70)	0.54 (0.65)	0.55 (0.66)	0.47 (0.64)	0.41 (0.59)
Emotional health	0.99 (0.85)	0.82 (0.81)	0.78 (0.80)	0.66 (0.76)	0.44 (0.69)
Self-care activities	0.57 (0.79)	0.42 (0.69)	0.41 (0.68)	0.39 (0.69)	0.26 (0.57)
Coping with MS	0.81 (0.83)	0.63 (0.74)	0.64 (0.77)	0.56 (0.76)	0.45 (0.70)
Carer relationships	0.55 (0.77)	0.52 (0.72)	0.58 (0.78)	0.50 (0.76)	0.43 (0.70)
Familial relationships	0.71 (0.82)	0.61 (0.79)	0.78 (0.86)	0.71 (0.84)	0.68 (0.82)
Living arrangements	0.59 (0.81)	0.52 (0.76)	0.50 (0.73)	0.51 (0.72)	0.56 (0.79)
Finances	0.36 (0.69)	0.41 (0.74)	0.35 (0.67)	0.29 (0.60)	0.21 (0.54)

	Disability severity			
	Nil (*n* = 399)	Mild (*n* = 340)	Moderate (*n* = 602)	Severe (*n* = 314)
Composite adversity	0.39 (0.59)	0.48 (0.58)	0.64 (0.69)	0.52 (0.67)
Emotional health	0.67 (0.74)	0.78 (0.77)	0.87 (0.83)	0.59 (0.79)
Self-care activities	0.26 (0.54)	0.32 (0.62)	0.53 (0.77)	0.44 (0.70)
Coping with MS	0.48 (0.69)	0.60 (0.72)	0.73 (0.80)	0.59 (0.79)
Carer relationships	0.32 (0.63)	0.47 (0.70)	0.67 (0.81)	0.57 (0.76)
Familial relationships	0.56 (0.76)	0.61 (0.77)	0.82 (0.88)	0.74 (0.86)
Living arrangements	0.36 (0.65)	0.49 (0.73)	0.60 (0.78)	0.59 (0.79)
Finances	0.30 (0.62)	0.31 (0.64)	0.41 (0.72)	0.28 (0.63)

For composite adversity	Sex and lockdown	
	No lockdown exposure	Lockdown exposure
Male	0.48 (0.63)	0.61 (0.64)
Female	0.43 (0.61)	0.83 (0.70)

All COVID-19-related adversity variables were represented on a three-point scale: (0) no adversity, (1) minor adversity, and (2) major adversity. For simplicity, only metropolitan lockdown was utilised in this table

**Fig. 1:**
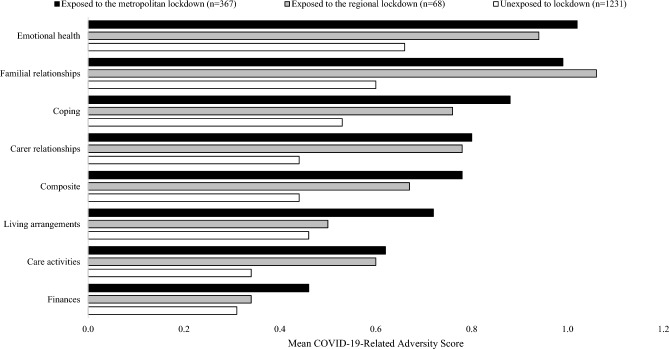
Bar chart of average COVID-19-related adversity scores stratified by exposure to the metropolitan and regional lockdowns. Note: Composite refers to an adversity measure comprising the arithmetic average of all other COVID-19-related adversity scores. COVID-19-related adversity was measured on a 0-2 integer scale. Differences in each group of scores were significant at the 0.01 level, according to Fisher’s Exact Test

Lastly, as illustrated in Fig. 1, stratification by lockdown exposure showed that mean COVID-19-related adversity rankings were larger for participants who were exposed to lockdown. To illustrate, in the domain of coping with MS the average adversity ranking for unexposed participants was 0.53, compared to 0.76 and 0.88 for participants exposed to the regional and metropolitan lockdowns, respectively (*p* < 0.01). Moreover, and as demonstrated in Table 3, females reported higher COVID-19-related adversity, on average, when exposed to lockdown (0.83 [females] compared to 0.61 [males]).

### Stratification of AQoL-8D HSUs and dimensional scores over COVID-19-related adversity variables and lockdown exposure

Stratifications of mean AQoL-8D HSUs over COVID-19-related adversity variables are presented in Table 4. Participants with composite adversity rankings zero, one, and two had mean HSUs of 0.685, 0.574, and 0.463, respectively. Notably, each reduction in HSU exceeded the 0.08 AQoL-8D MID and the mean HSU values were well below the 0.80 population norm. In contrast with the previous results, stratifying HSU by lockdown exposure revealed no relationships.

**Table 4:** Frequency and percentage of participants exposed to lockdown or reporting composite and dimension-specific COVID-19-related adversity, and their mean AQoL-8D health state utilities (HSUs)

	Frequency, (%)	Mean (SD) AQoL-8D HSU
Lockdown exposure		
0 (No exposure)	1231 (73.9)	0.627 (0.215)
1 (Regional exposure)	68 (4.1)	0.605 (0.202)
2 (Metropolitan exposure)	367 (22.0)	0.631 (0.224)
Composite adversity		
0 (No adversity)	933 (56)	0.685 (0.203)
1 (Minor adversity)	588 (35.3)	0.574 (0.196)
2 (Major adversity)	145 (8.7)	0.463 (0.197)
Emotional health		
0 (No adversity)	784 (47.1)	0.672 (0.211)
1 (Minor adversity)	506 (30.4)	0.637 (0.201)
2 (Major adversity)	376 (22.6)	0.519 (0.196)
Self-care activities		
0 (No adversity)	1179 (70.8)	0.668 (0.205)
1 (Minor adversity)	292 (17.5)	0.564 (0.204)
2 (Major adversity)	195 (11.7)	0.476 (0.180)
Coping with MS		
0 (No adversity)	930 (55.8)	0.680 (0.205)
1 (Minor adversity)	448 (26.9)	0.595 (0.207)
2 (Major adversity)	288 (17.3)	0.507 (0.189)
Carer relationships		
0 (No adversity)	1051 (63.1)	0.677 (0.204)
1 (Minor adversity)	353 (21.2)	0.562 (0.199)
2 (Major adversity)	262 (15.7)	0.516 (0.200)
Familial relationships)		
0 (No adversity)	898 (53.9)	0.670 (0.207)
1 (Minor adversity)	367 (22.0)	0.615 (0.203)
2 (Major adversity)	401 (24.1)	0.541 (0.204)
Living arrangements		
0 (No adversity)	1059 (63.6)	0.665 (0.209)
1 (Minor adversity)	351 (21.1)	0.594 (0.197)
2 (Major adversity)	256 (15.4)	0.513 (0.202)
Finances		
0 (No adversity)	1282 (77.0)	0.648 (0.207)
1 (Minor adversity)	199 (11.9)	0.592 (0.210)
2 (Major adversity)	185 (11.1)	0.506 (0.216)

Therefore, it may be observed in the preceding results that lockdown is associated with COVID-19-related adversity, which is in turn associated with reduced HSUs. However, no direct relationship was identified between lockdown exposure and HSU. This implies that COVID-19-related adversity mediates the relationship between lockdown exposure and health-related quality of life.

### Proportions of participants reporting COVID-19-related adversity and reduced HSU

Table 4 also displays the numbers of participants who reported each level of dimension-specific or composite COVID-19-related adversity. Importantly, it shows that 44.0% (*n* = 733) of participants reported minor or major adversity, on average, across all the measured domains of health (represented by the composite measure). Additionally, 74.3% (*n* = 1238) of participants reported a minimum of minor adversity in at least one dimension of health. In contrast, only 13.9% (*n* = 232) of participants reported benefits in at least one health dimension.

### Thematic analysis of qualitative data

**Fig. 2:**
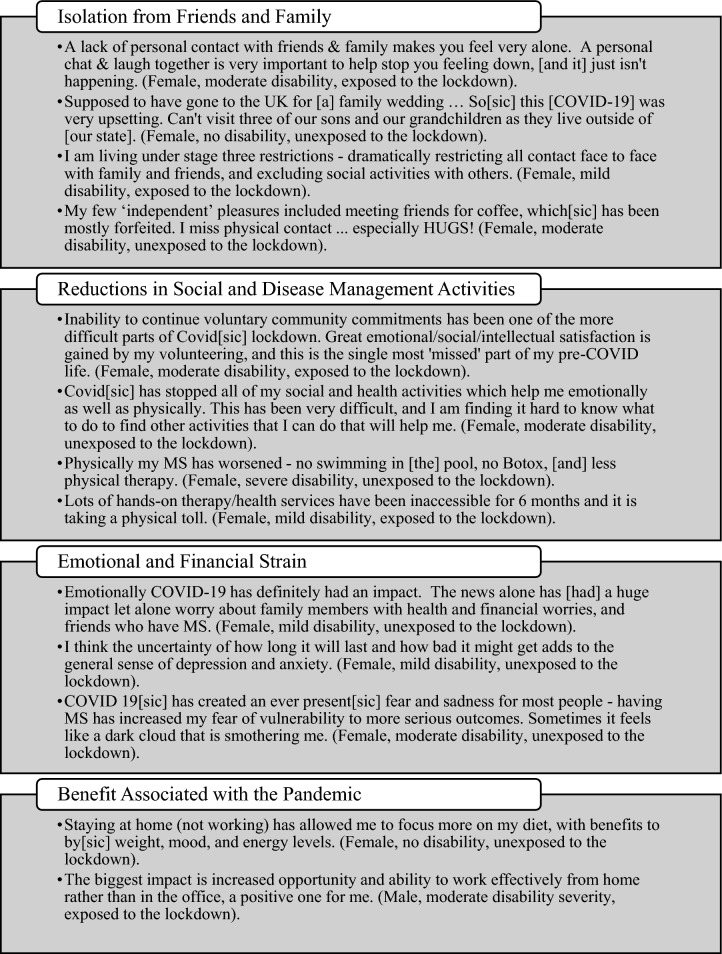
Verbatim quotes supporting themes of isolation from friends and family, reduction in social and disease management activities, financial and emotional strain, and benefit

Qualitative data are provided in Fig. 2 (supported by Supplementary Table 2) as selected, verbatim quotations (with adjustments marked by square brackets). In this study, 30.6% (*n* = 509) of participants who responded to the 2020QoL also provided qualitative data. Thematic analysis established that the core theme was that people living with MS had a negative experience of the pandemic and lockdown. Major subthemes reinforcing the core theme included: isolation from friends and family, reductions in social and disease management activities, and emotional and financial strain. Contrasting with the core theme, some people living with MS said that they benefited from the pandemic and lockdowns. These participants indicated that they benefited from being able to focus on personal issues or work from home.

### Factors associated with perceptions of greater COVID-19-related adversity

Table 5 summarises the results of the multivariable ordered probit regressions that assessed how sociodemographic and clinical factors influenced composite and emotional health-related COVID-19-related adversity rankings (results pertaining to all other COVID-19-related adversity variables are located in Supplementary Table 3). The ordered probit, conducted for composite adversity, revealed two important associations. It indicated that relapse-onset MS phenotypes (0.165, *p* < 0.05) and greater disability severity (mild disability: 0.177 (*p* < 0.05), moderate disability: 0.425 (*p* < 0.01), severe disability: 0.337 (*p* < 0.01)) were positively associated with reports of COVID-19-related adversity.

**Table 5:** Results of multivariable ordered probit regressions for the composite and emotional health COVID-19-related adversity variables

Results are displayed in the format: coefficient (95% CI)	Composite		Emotional health		Composite (Sex × Lockdown interaction)	
Age (years)	−**0.013**	**(**−**0.018, **−**0.008)** ***	−**0.016**	**(**−**0.024, **−0.009)***	−**0.013**	**(**−**0.018, **−0.007)***
Sex (female)	0.020	(−0.098, 0.139)	**0.143**	(0.001, 0.285)**	−0.124	(−0.265, 0.017)*
Employment (employed)	-0.126	(−0.256, 0.005)*	−0.048	(−0.192, 0.096)	−0.126	(−0.259, 0.006)*
Phenotype (relapse-onset)	**0.165**	(0.011, 0.319)**	**0.281**	(0.065, 0.497)**	**0.171**	(0.013, 0.329)**
Education						
(1) Occupational	−0.050	(−0.102, 0.202)	0.122	(−0.059, 0.302)	−0.090	(−0.066, 0.246)
(2) Bachelor’s degree	−0.062	(−0.244, 0.120)	0.129	(−0.066, 0.323)	−0.032	(−0.217, 0.152)
(3) Postgraduate degree	0.038	(−0.144, 0.220)	**0.223**	(0.033, 0.414)**	0.081	(−0.099, 0.261)
Disability severity						
(1) Mild	**0.177**	(0.035, 0.320)**	**0.192**	(0.052, 0.333)***	**0.204**	(0.055, 0.354)***
(2) Moderate	**0.425**	(0.272, 0.577)***	**0.352**	(0.191, 0.512)***	**0.455**	(0.302, 0.609)***
(3) Severe	**0.337**	(0.159, 0.516)***	−0.049	(−0.309, 0.211)	**0.360**	(0.180, 0.540)***
Socioeconomic area						
(1) Quartile two	0.049	(−0.147, 0.245)	−0.010	(−0.068, 0.359)	0.019	(−0.113, 0.151)
(2) Quartile three	-0.073	(−0.259, 0.114)	−0.007	(−0.018, 0.427)	−**0.196**	**(**−**0.341, **−0.050)***
(3) Quartile four	0.151	(−0.038, 0.339)	0.204	(−0.232, 0.217)	−0.099	(−0.252, 0.053)
(4) Quartile five	0.138	(−0.048, 0.323)	0.146	(−0.243, 0.223)	−0.123	(−0.289, 0.063)
Lockdown						
(1) Metropolitan	**0.479**	(0.354, 0.603)***	**0.440**	(0.273, 0.607)***	0.147	(−0.106, −0.399)
(2) Regional	**0.435**	(0.225, 0.645)***	**0.445**	(0.121, 0.769)***	−0.033	(−0.505, −0.439)
Sex (female) × Lockdown						
(1) Female × Metro. lock					**0.390**	(0.110, 0.670)***
(2) Female × Regio. lock					0.523	(−0.023, 1.070)*
Cut one	−0.044	(−0.470, 0.382)	−0.200	(−0.736, 0.337)	−0.235	(−0.676, 0.205)
Cut two	0.990	(0.557, 1.423)	0.656	(0.141, 1.171)	0.842	(0.408, 1.276)

Bolding denotes significance at the *α* = 0.05 level or less. Asterisks denote the following levels of significance: * significant at the *α* = 0.10 level, ** significant at the *α* = 0.05 level, and *** significant at the *α* = 0.01 level. Measures of significance do not apply to cut-points. Coefficients in the table represent sentiment associated with higher adversity rankings, and the cut-points represent levels of sentiment that must be reached before a participant is likely to report a higher level of adversity. If cut one is exceeded, a participant is most likely to report minor adversity. Similarly, if cut two is exceeded a participant is most likely to report major adversity

Crucially, exposure to the metropolitan lockdown was strongly and consistently associated with higher adversity perceptions, both dimension-specific and composite. Evidencing this, the effect size on the metropolitan lockdown indicator was relatively large in the regression for composite adversity (0.479, *p* < 0.01). Also of interest, a positive interaction was observed between sex and metropolitan lockdown exposure. That the coefficient for males became insignificant after adjustment for this interaction should be analysed with caution due to the relatively small number of males (*n* = 336 [20.2%]) present in the study cohort.

Additionally, Supplementary Table 4 provides the results of the sensitivity analysis regarding which participants were considered exposed to the regional lockdown. This analysis demonstrated that results were robust to this decision.

### HSU values representing changes in HRQoL associated with COVID-19-related adversity

Table 6 displays the results of univariable and multivariable regressions of the COVID-19-related adversity variables on HSU, demonstrating strong associations. To illustrate, we estimated that participants ranked as experiencing minor or major adversity, on the composite adversity scale, had their HSUs reduced by −0.110 and −0.221 utility points, on average (*p* < 0.01). These associations persisted after controlling for clinical and sociodemographic confounders and exceeded the MID (0.08). Importantly, no significant association between lockdown exposure and HSU was observed.

**Table 6:** Associations between AQoL-8D health state utilities, and COVID-19-related adversity and lockdown exposure

	Univariable regressions using lockdown exposure and individual COVID-19 adversity variables, separately		Multivariable regressions using lockdown exposure and individual COVID-19 adversity variables, separately^c^		Single model, multivariable regression including all dimensional COVID-19 adversity variables^c^		Logit regressions with clinically meaningful change (> 0.08) in health state utility as the outcome^d^	
Lockdown								
Metropolitan	0.003	(−0.022, 0.028)	−0.002	(−0.023, 0.019)				
Regional	−0.021	(−0.073, 0.031)	−0.018	(−0.061, 0.024)				
Composite adversity								
Mild adversity	−**0.110**	**(**−**0.131, **−0.090)***	−**0.089**	**(**−**0.106, **−0.072)***			**1.784**	(1.301, 2.446)***
Major adversity	−**0.221**	**(**−**0.257, **−0.186)***	−**0.166**	**(**−**0.197, **−0.135)***			**2.716**	(1.680, 4.391)***
Emotional health								
Mild adversity	−**0.035**	**(**−**0.058, **−0.012)***			−0.020	(−0.040, 0.001)*	1.084	(0.756, 1.555)
Major adversity	−**0.152**	**(**−**0.177, **−0.127)***			−**0.061**	**(**−**0.089, **−0.034)***	**2.307**	(1.620, 3.287)***
Self-care activities								
Mild adversity	−**0.104**	**(**−**0.130, **−0.078)***			−**0.029**	**(**−**0.053, **−0.005)**	1.349	(0.914, 1.991)
Major adversity	−**0.188**	**(**−**0.219, **−0.157)***			−**0.037**	**(**−**0.069, **−0.006)**	**2.228**	(1.465, 3.386)***
Coping								
Mild adversity	−**0.085**	**(**−**0.107, **−0.062)***			−**0.035**	**(**−**0.058, **−0.012)***	1.171	(0.825, 1.663)
Major adversity	−**0.172**	**(**−**0.198, **−0.145)***			−**0.039**	**(**−**0.071, **−0.007)**	**1.851**	(1.270, 2.697)***
Carer relationships								
Mild adversity	−**0.115**	**(**−0.140, − 0.091)***			−**0.027**	**(**−**0.051, **−0.003)**	**1.715**	(1.200, 2.452)***
Major adversity	−**0.160**	**(**−0.187, − 0.132)***			−0.021	(−0.050, 0.009)	**1.938**	(1.316, 2.853)***
Familial relationships								
Mild Adversity	−**0.056**	**(**−**0.081, **−0.031)***			−0.003	(−0.024, 0.019)	**1.654**	(1.147, 2.387)***
Major adversity	−**0.130**	**(**−**0.154, **−0.106)***			−0.008	(−0.034, 0.018)	**1.839**	(1.297, 2.609)***
Living arrangements								
Mild adversity	−**0.071**	**(**−**0.096, **−0.047)***			0.015	(−0.007, 0.037)	1.307	(0.899, 1.901)
Major adversity	−**0.152**	**(**−**0.180, **−0.124)***			−0.006	(−0.037, 0.024)	**1.849**	(1.256, 2.723)***
Finances								
Mild adversity	−**0.057**	**(**−**0.089, **−0.026)***			−**0.032**	**(**−**0.058, **−0.007)**	0.770	(0.467, 1.270)
Major adversity	−**0.131**	**(**−**0.163, **−0.099)***			−**0.035**	**(**−**0.065, **−0.005)**	1.289	(0.821, 2.022)

Results are displayed in the format: coefficient (95% CI). Bolding denotes significance at the *α* = 0.05 level or less. Asterisks denote the following levels of significance: * significant at the *α* = 0.10 level, ** significant at the *α* = 0.05 level, and *** significant at the *α* = 0.01 level. ^c^ Regressions were adjusted for all sociodemographic and clinical factors. ^d^ Results are presented as odds ratios

Simultaneously regressing all dimension-specific COVID-19-related adversity variables on HSU, while controlling for confounders, revealed that major emotional adversity had the largest impact on HSU (−0.061, *p* < 0.01, Table 5). In addition, no clinically significant relationships were identified between reports of COVID-19-related benefits and HRQoL, as evidenced in Supplementary Table 1.

### Associations between COVID-19-related adversity and clinically meaningful reductions in HSU

Table 6 also displays the results of the logistic regressions. Overall, they showed that reports of COVID-19-related adversity were associated with clinically significant reductions in HSU. For example, participants ranked as experiencing minor and major adversity on the composite scale were 1.784 and 2.716 times more likely to experience a clinically significant decrease in HSU (*p* < 0.01).

## Discussion

Our study was the first to use the comprehensive AQoL-8D MAUI, a specialised COVID-19 questionnaire, and supplementary qualitative data to evaluate the health economic impact of the COVID-19 pandemic and lockdowns on people living with MS. We identified a strong and positive association between lockdown exposure and higher COVID-19-related adversity rankings. In turn, reports of COVID-19-related adversity were found to significantly reduce participant HSUs, and thus diminish their HRQoL. Our logit regressions, which controlled for unobserved, time-invariant factors, supported this finding. They indicated that participants reporting COVID-19-related adversity were more likely to experience a clinically meaningful reduction in HSU during the pandemic. Themes in qualitative data also supported our findings. Importantly, our study generated HSU values that can be applied in health economic models.

### Associations between COVID-19-related adversity and participant characteristics

Higher COVID-19-related adversity rankings were found to have a strong, positive, and statistically significant association with metropolitan lockdown exposure. This result was supported by an Australian general population study (*n* = 1599) which determined that deteriorations in mental and physical health were associated with exposure to COVID-19-related lockdowns [23]. We also identified that females were more likely to report COVID-19-related adversity when exposed the lockdown, possibly because they experience greater psychological distress [24]. However, in our study exposure to lockdown was only a component cause, with other factors contributing substantially to greater perceptions of COVID-19-related adversity.

Most important among these factors was higher MS-related disability severity. In a British study of people living with chronic pain (*n* = 519), a similar relationship between disability severity and COVID-19-related adversity was demonstrated [25]. Participants with higher MS-related disability reporting greater COVID-19-related adversity may be explained by these individuals being more reliant on health services [26]. Consequently, COVID-19-related restrictions on healthcare accessibility would have impacted them the most.

### Associations between HSU and COVID-19-related adversity

Significant, negative associations between HSU (and thus HRQoL) and COVID-19-related adversity were identified. For example, multivariable regression demonstrated that participants ranked as perceiving minor and major COVID-19-related adversity (on the composite scale) experienced mean reductions in HSU of 0.089 and 0.166, respectively. Logits produced congruent results, indicating that these same participants were 1.78 and 2.72 times more likely to experience a reduction in HSU exceeding the 0.08 MID, before to during the pandemic. Supporting this, an Australian general population study (*n*≈1900) also identified a substantial reduction in HRQoL between before and during pandemic study samples [27]. Additionally, 44.0% of participants were categorised as experiencing minor or major adversity on the composite scale, and 34.0% were identified as having experienced a clinically meaningful reduction in HSU. As such, COVID-19-related adversity can be said to have significantly affected a substantial proportion of Australian people living with MS.

### The association between exposure to the lockdown and HRQoL

The association between lockdown exposure and HRQoL is negative and mediated through the COVID-19-related adversity variables. No direct relationship could be identified between lockdown exposure and HSU. An explanation for this may be that participants outside of lockdown also perceived COVID-19-related adversity, thus obfuscating the relationship between lockdown exposure and decremented HRQoL.

### Benefits associated with the COVID-19 pandemic and lockdowns

13.9% of study participants reported COVID-19-related benefits, a result supported by the literature [23, 28]. However, we did not find clinically significant relationships between COVID-19-related benefits and HSU. Consequently, we concluded that the lockdown and pandemic were not responsible for systematic improvements in HRQoL.

### Key dimensions of health through which COVID-19-related adversity reduced HRQoL

For people living with MS, pandemic-related reductions in HSU were most strongly associated with COVID-19-related adversity in the domains of emotional wellbeing and maintenance of self-care activities. This conclusion was based on emotional wellbeing and maintenance of self-care activities being consistently significant in regression (with relatively large coefficients), as well as highlighted in the thematic analysis of qualitative data. Three other MS-specific studies also identified that the mental health of their participants was substantially impacted by the pandemic and lockdowns [29–31], as did six general population studies [32–37]. Existing non-MS-related studies also supported our results regarding self-care activities [35, 38].

### Translational impact of generated HSU values in health economics models

The HSUs associated with the COVID-19-related adversity rankings generated in our study can be used to populate health economics models [39]. In particular, they could be used in models to evaluate the cost-effectiveness of lockdowns from the perspective of people living with MS (or persons with other complex and chronic neurological diseases) [40, 41]. They could also be used in models intended to determine the viability of alternative modes of delivery of care to people living with MS during pandemics and lockdowns, including remote/telehealth delivery [26]. The above implications apply both to regression estimates of HSU decrements and mean HSUs obtained through stratification. Furthermore, the percentages of participants reporting COVID-19-related adversity or clinically important reductions in HSU, in this study, could be used to generate approximations for health economic analyses and simulations.

### Strengths and limitations

A key strength of the study is that it used the AQoL-8D MAUI, which is particularly sensitive to changes in psychosocial health and effectively captures subjective wellbeing. The AQoL-8D has also been identified as having preferential sensitivity for our study population in validation studies [3, 5]. Another important strength of our study is that it obtained data from the Australian Multiple Sclerosis Longitudinal Study cohort, a large and representative sample of Australian people living with MS [14]. This provided both statistical power and internal validity to the study’s findings.

A key limitation of the paper was that we could not control for the effect of COVID-19 infection, although this is unlikely to have confounded results. This is because there was a relatively low incidence rate in Australia (including areas that were in lockdown) and participants did not indicate any infections in free-text responses [42]. Additionally, it is difficult to determine how generalisable results will be to other MS and chronic disease populations, especially those in non-high income and non-anglophonic countries.

### Conclusions

A key and practical product of our study was the estimation of HSUs associated with COVID-19-related adversity for a representative cohort of people living with MS. These values can be applied in health economics models relating to people living with MS (and other persons with chronic and complex diseases, especially neurological diseases) and further COVID-19 outbreaks or future pandemics. Such models could assist policymakers in evaluating the implications of lockdowns and the viability of measures intended to ameliorate their impacts and the effects of pandemics. Overall, our findings should raise awareness of the effects of pandemics and isolation quarantines on populations with complex and chronic diseases and encourage a proactive response.

## Supplementary Information

Below is the link to the electronic supplementary material.

Supplementary file1 (DOCX 351 kb)
