# Supplementary material for: The quality of life impact of the COVID-19 pandemic and lockdowns for people living with multiple sclerosis (MS): evidence from the Australian MS Longitudinal Study
Source: Qual Life Res. 2024 Apr 5;33(6):1675–89. doi: 10.1007/s11136-024-03620-4 (PMC11116258; doi:10.1007/s11136-024-03620-4)
Supplement: Supplementary file 1 — Supplementary file1 (DOCX 351 kb) [file 11136_2024_3620_MOESM1_ESM.docx]

**The quality of life impact of the COVID-19 pandemic and lockdowns for people living with Multiple Sclerosis (MS): evidence from the Australian MS Longitudinal Study**

Quality of Life Research

Glen J. Henson ^1^, Ingrid van der Mei ^1^, Bruce V. Taylor ^1^, Paul Blacklow ^2^, Suzi B. Claflin ^1^, Andrew J. Palmer ^1^, Carol Hurst ^1^, and Julie A. Campbell ^1^

^a^ Menzies Institute for Medical Research (University of Tasmania), 17 Liverpool St, Hobart, Tasmania, Australia, 7000.

^b^ Tasmanian School of Business and Economics (University of Tasmania), Churchill Avenue, Sandy Bay, Tasmania, Australia, 7005.

Corresponding Author

Julie A. Campbell: julie.campbell@utas.edu.au, ORCID 0000-0002-1820-6758.

**Supplementary Materials**

**Table of Contents**

| **Title** | **Description** | **Page** |
| --- | --- | --- |
| *Methods Appendices* |  |  |
| Appendix 1 | Explanations regarding data sources, a summary of missing data, and specifics of imputation | 2 |
| Appendix 2 | Composite adversity scores and defining lockdown exposure | 3 |
| Appendix 3 | Information on the ordinal probits and logits used in this study | 4 |
| *Supplementary Figure* |  |  |
| Supplementary Figure 1 | Image of the COVID-19 questionnaire used to elicit adversity rankings | 6 |
| *Supplementary Tables* |  |  |
| Supplementary Table 1 | Analysis of the health-related quality-of-life impact attributable to perceptions of COVID-19-related benefit | 7 |
| Supplementary Table 2 | Verbatim quotations evidencing themes in qualitative data | 8 |
| Supplementary Table 3 | Further results from ordered probits regressions of COVID-19-related adversity variables | 12 |
| Supplementary Table 4 | Sensitivity analysis for the lockdown variable, category two, using composite adversity as an outcome measure | 13 |
| References |  | 14 |

**Methods Appendices**

**Appendix 1 – Additional data sources, missing data, and imputation**

*Additional data sources*

In addition to AQoL-8D health state utilities, COVID-19-related adversity scores, and free text data, the 2020QoL extracted data regarding disability severity, age, sex, MS phenotype (type of MS), and index of relative socioeconomic advantage and disadvantage (IRSAD) and lockdown status (via participant-provided postcodes). Additional, cross-sectional clinical and sociodemographic data were obtained from the 2018 Medication and Disease Course Survey (October 2018 – December 2018, 2678 invited and 1740 [66.8%] responded), the 2019 Medication and Disease Course Survey (October 2019 – December 2019, 2537 invited and 1767 [69.6%] responded) and the 2020 Disease Course Survey (October 2020 – December 2020, 2537 invited and 1604 [62.3%] responded).

The Disease Course Surveys assessed disease outcomes, use of disease-modifying therapies, and various clinical and sociodemographic variables. Education level, MS phenotype, and employment status were obtained from these surveys. Employment status was extracted only from the 2020 Disease Course Survey, due to the dynamism of the variable. Being relatively time-invariant, education level and MS phenotype were sourced from all three surveys to minimise missing data.

Data for use in the longitudinal analysis were obtained from the 2019 Disease Course Survey, the 2020 Quality of Life Survey, and the 2020 Sleep and Nurses Survey (February 2020 – March 2020, 2496 participants invited, 1722 (69.0%) responded). Specifically, before and during pandemic health state utilities (HSUs) were acquired from the Sleep and Nurses Survey and Quality of Life Survey, respectively, with additional disability severity data being sourced from the 2019 Disease Course Survey. 1338 (77.7%) of respondents to the 2020 Sleep and Nurses Survey also responded to the 2020 Quality of Life Survey, with 1148 (85.8%) of these participants responding also to the 2019 Disease Course Survey. Only data pertaining to these subsets of participants could be utilised in longitudinal analysis. Additionally, of the 1722 participants who responded to the 2020 Sleep and Nurses Survey, 97 (5.6%) did so following the initial nationwide COVID-19 lockdown, initiated on 23^rd^ March 2020 as the presence of the pandemic was recognised in Australia. Participants responding after this date were not excluded from the study, however, due to the brief time they had been in lockdown.

*Summary of missing data indicating minimal missing data*

| Variable | Missing Observations |
| --- | --- |
| Emotional health | 7 |
| Self-Care activities | 3 |
| Coping | 15 |
| Carer relationships | 16 |
| Family relationships | 11 |
| Living arrangements | 3 |
| Finances | 5 |
| HSU | 14 |
| Employment Status | 343 |
| MS Phenotype | 140 |
| Education Level | 11 |
| Disability Severity | 11 |
| Socioeconomic area | 1 |

Each variable had 1666 observations associated with it.

Variables not appearing in the above table had no missing data.

*Hot deck imputation*

Hot-deck imputation involves replacing participants’ missing data with the data of participants who responded similarly to mutually completed COVID-19 questions. The latter participants are selected randomly to minimise bias. Before any imputation was conducted, 17 participants who did not respond ≥3 of the COVID-19 questions were excluded from the study, as there was insufficient data to effectively impute their missing values.

*Multiple imputation by chained equations*

To rectify missingness in sociodemographic, clinical, and outcome variables, multiple imputation by chained equations was applied [1]. This method performs well when imputing categorical variables, making it appropriate in the context of this study [2]. Moreover, data generated by multiple imputation are representative of uncertainty in the imputation process. As such, should an imputation equation be poorly specified, the range of imputed values for each missing observation will be wide. In regression, this is represented by wider confidence intervals [3]. In addition, this study’s multiple imputation equations used all non-adversity variables as regressors, and 20 values were generated per missing observation.

**Appendix 2 – Composite adversity scores and defining lockdown exposure**

*Composite adversity scores*

To represent composite adversity on a three-point Likert scale, participant average COVID-19-related adversity scores were rounded to the nearest integer values. For example, a participant reporting major pandemic-related adversity in two relevant health dimensions, and minor adversity in the remaining two, would score 1.50 on the composite adversity measure. This participant’s simplified score is 2.00, after rounding to the nearest integer value. Therefore, a ranking of major, composite adversity should be interpreted as a participant perceiving major adversity, on average, across the relevant dimensions of health.

*Defining lockdown exposure*

This study acknowledged that the remainder of Victoria entered stage three lockdown from August 5^th^ (hereon referred to as “the regional lockdown”, with simultaneous references to both lockdowns not possessing preceding adjectives). Based on differences in duration and severity of exposure – the regional lockdown began 29 days after the metropolitan lockdown, and was not escalated to stage four – inclusion of participants affected by the regional lockdown ($n=68$) in the exposed category was considered inappropriate. A third category labelled *regional* was established to account for these participants.

This study assumed that a minimum of one week of exposure to the regional lockdown was necessary for significant exposure to occur. Thus, respondents who were not exposed to the regional lockdown for at least one week ($n=18$) were considered unexposed. To test this assumption, sensitivity analysis was undertaken (results displayed in Supplementary Table 2). This analysis involved replacing the original regional lockdown category with two others. One applied only to participants exposed for greater than two weeks ($n=44$), and the other applied to any participant exposed to the regional lockdown ($n=86$), regardless of duration. Regressions conducted using these categories did not generate substantial variation in coefficient estimates. Additionally, due to uncertainty surrounding the specification of the regional lockdown category, combined with relatively few, relevant observations ($n=68$ versus $n=367$ for the metropolitan lockdown).

**Appendix 3 – Ordered probits and longitudinal regression analysis**

*Ordered probits*

As stated in the main text, ordered probits estimate coefficients that represent sentiment toward dependent variable rankings. Cut points are levels of sentiment at or above which an individual will select a particular rank. In explanation, consider the COVID-19-adversity variable $Q_{i}$, which can take the values:

$$Q_{i}=\left\{ \begin{aligned} 2 \left( Major Adversity \right) \\ 1 \left( Minor Adversity \right) \\ 0 \left( No Adversity \right) \end{aligned} \right.$$

A participant will only select rank one when $\theta_{1}\leq Sentiment<\theta_{2}$, where $\theta_{1}$ represents the cut point between no adversity and minor adversity, and $\theta_{2}$ represents the cut point between minor adversity and major adversity [4]. Therefore, a participant will select a specific rank when sentiment levels are not sufficiently high or low to place them in an adjacent category. If $\mathbf{Y}$ is a $16\times1$ vector of cross-sectional regressors (where 1 is the number of observations per variable and 15 [plus the constant] is the number of variables), $\boldsymbol{\rho}$ is a $1\times16$ vector of estimated parameters and $e_{i}^{B}$ are model residuals, such that:

$$P\left( Q_{i}=0,1,2 \right)=\boldsymbol{\rho}\mathbf{Y}_{i}\mathbf{+}e_{i}^{B}$$

The function of an ordered probit for $\mathrm{CO}M_{i}=1$ can be mathematically summarised as [4]:

$$P\left( \mathrm{COM}_{i}=1 \right)=P\left( \theta_{1}<\boldsymbol{\rho}\mathbf{Y}_{i}\mathbf{+}e_{i}\leq\theta_{2} \right)=P\left( \theta_{1}-\boldsymbol{\rho}\mathbf{Y}_{i}<e_{i}\leq\theta_{2}-\boldsymbol{\rho Y}{}_{i} \right)$$

$=CDF\left( \theta_{2}-\boldsymbol{\rho}\mathbf{Y}_{i} \right)-CDF\left( \theta_{1}-\boldsymbol{\rho}\mathbf{Y}_{i} \right)$

Where $P\left( \mathrm{COM}_{i}=1 \right)$ is the probability of the ith individual selecting rank one (minor adversity), and CDF is the normal cumulative distribution function. Ordered probit models were used in this study to determine which factors contributed to sentiment toward higher COVID-19-related adversity rankings. These models were generally specified as:

$$P\left( Q_{\mathrm{ij}}=0,1,2 \right)=\boldsymbol{\rho}\mathbf{Y}_{i}+e_{\mathrm{ij}}^{B}$$

Where $Q_{\mathrm{ij}}$ is the ranking associated with the ith individual and the jth COVID-19-related adversity question.

Evidence of heteroscedasticity was detected in most of the ordered probit regressions, excluding regressions for adversity associated with living arrangements and finances. Heteroscedastic consistent ordered probits (hereon referred to as “hetoprobits”) were estimated to compensate for heteroscedasticity [5]. These models use auxiliary scedastic regressions to estimate the variance of the error term as a multiplicative function of regressors. Explanatory variables were selected for the auxiliary regressions based on: (1) the magnitude of the change in results that their inclusion elicited (an indication of bias in the unadjusted model); and (2) statistical significance in the auxiliary regressions. Disability severity was frequently identified as a variable responsible for heteroscedasticity in adversity perceptions.

*Logits*

The longitudinal dependent variable (a binary indicator designated $\Delta HSU_{i}$) was applied in logistic regressions, which took the (linearised) form:

$$\Delta HSU_{i}=\gamma_{0}+\gamma_{1j}Q_{\mathrm{ij}}+\gamma_{2j}\Delta EDSS_{i}+\Delta e_{\mathrm{ij}} (1)$$

Where $\gamma_{0}$ represents baseline likelihood, $Q_{\mathrm{ij}}$ is the $\mathrm{jth}$ COVID-19-related adversity variables, $\Delta EDSS_{i}$ the change in disability severity, $\gamma_{1j}$ and $\gamma_{2j}$ the respective coefficients, and $e_{\mathrm{ij}}$ the residuals. Note that adversity variables were trialled individually due to high multicollinearity affecting variable significance in multivariable regression. Time invariant regressors ($\mathrm{AG}E_{i}$, $\mathrm{SE}X_{i}$, $\mathrm{TYP}E_{i}$) and regressors exhibiting low variation ($\mathrm{WOR}K_{i}$, $\mathrm{ED}U_{i}$, $\mathrm{SEIF}A_{i}$) were excluded from the models. In explanation, consider a model where the $\mathrm{ith}$ individual has the health state utility $\mathrm{HS}U_{\mathrm{it}}$ at $t=1,2$, such that:

$$\mathrm{HS}U_{i1}=\gamma_{01j}+\gamma_{1j}^{*}Q_{i1j}+\gamma_{2j}^{*}\mathrm{EDS}S_{i1}+\boldsymbol{\gamma}_{\mathrm{Ij}}\boldsymbol{\varphi}_{i}+v_{\mathrm{ij}}+e_{i1j} (2)$$

$$\mathrm{HS}U_{i2}=\gamma_{02j}+\gamma_{1j}^{*}Q_{i2j}+\gamma_{2j}^{*}\mathrm{EDS}S_{i2}+\boldsymbol{\gamma}_{\mathbf{I}j}\boldsymbol{\varphi}_{i}+v_{\mathrm{ij}}+e_{i2j} (3)$$

Where $\gamma_{01j}$ and $\gamma_{02j}$ are baseline HSUs at their respective time-points (potentially different due to determinants such as seasonal variation), $\boldsymbol{\varphi}$ is an $S\times1$matrix of time-invariant regressors (where 1 is the number of observations per variable and S is the number of variables), $\boldsymbol{\gamma}_{\mathrm{Ij}}$ is a $1\times S$ vector of coefficients, and $v_{\mathrm{ij}}$ represents time-invariant between-participant heterogeneity. To generate an equation analogous to $(1)$, $(2)$ must be subtracted from $(3)$:

$$\left( \mathrm{HS}U_{i2}-HSU_{i1} \right)=\left( \gamma_{01j}-\gamma_{02j} \right)+\gamma_{1j}^{*}\left( Q_{i2j}-Q_{i1j} \right)+\gamma_{2j}^{*}(EDSS_{i2}-EDSS_{i1})-+\boldsymbol{\gamma}_{\mathrm{Ij}}\left( \boldsymbol{\varphi}_{i}-\boldsymbol{\varphi}_{i} \right)+\left( v_{\mathrm{ij}}-v_{\mathrm{ij}} \right)+\left( e_{i2j}-e_{i1j} \right)$$

$\Delta HSU_{i}^{*}=\gamma_{0j}^{*}+\gamma_{1j}^{*}\Delta Q_{\mathrm{ij}}+\gamma_{2j}^{*}\Delta EDSS_{i}+{\Delta e}_{\mathrm{ij}}^{*} (4)$

Where $\Delta Q_{ij}=Q_{ij}$, given that COVID-19-related adversity cannot be present before the pandemic reached Australia and $\Delta HSU_{i}^{*}$ is the non-dichotomised change in HSU. As illustrated, the effect of the time-invariant variables was cancelled in the difference model, disallowing their inclusion [6]. This is also why the logistic regressions could be used to estimate the association between health state utility and COVID-19-related adversity in the absence of potential confounding by unobserved, time-invariant factors.

**Supplementary Figure**

**
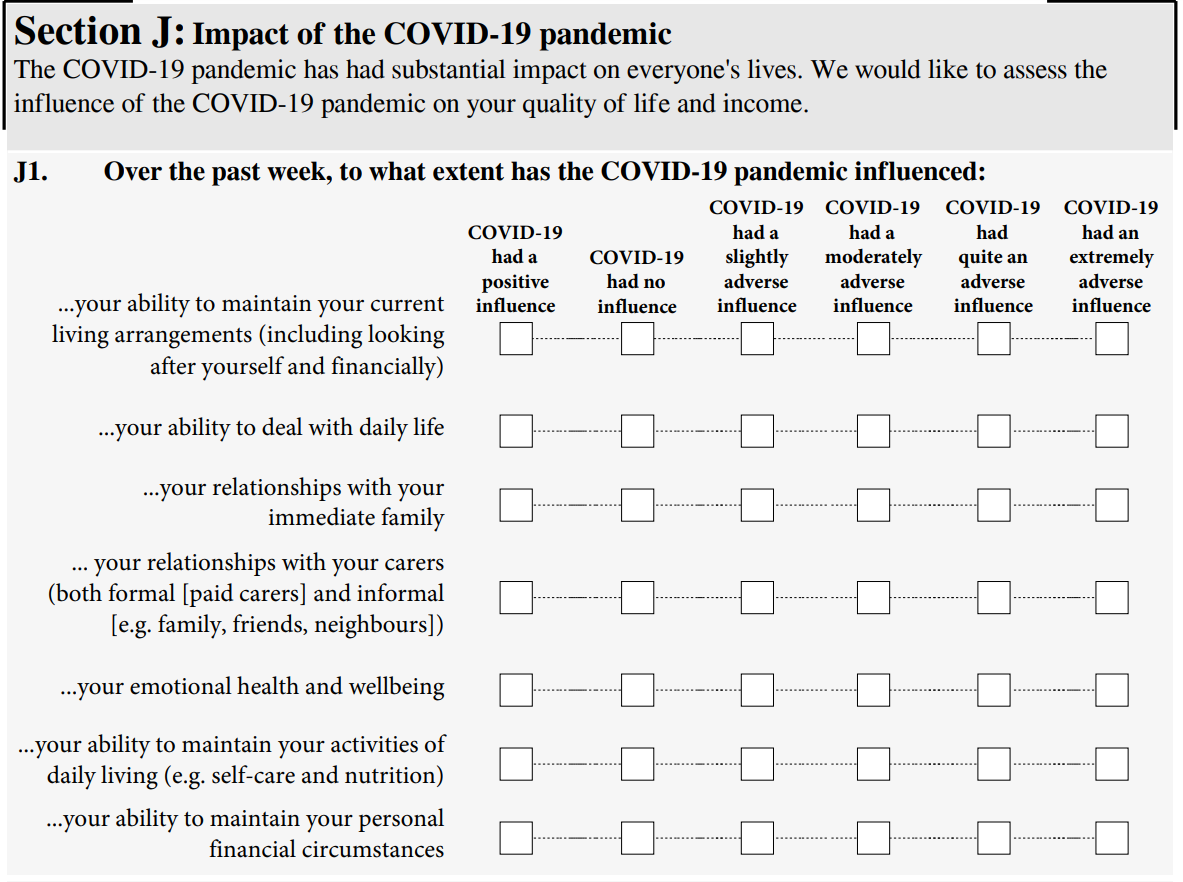
Supplementary Fig. 1** Image of the COVID-19 questionnaire embedded in the Australian Multiple Sclerosis Longitudinal Study 2020 Quality of Life survey

**Supplementary Tables**

**Supplementary Table 1** Analysis of the health-related quality-of-life impact attributable to perceptions of COVID-19-related benefit

|  | $\mathrm{HS}U_{i}$ attributable to perceptions of COVID-19-related benefit | | Proportions of participants reporting benefit, %(n) |
| --- | --- | --- | --- |
| Emotional health | 0.001 | (-0.043, 0.043) | 3.8 (63) |
| Care activities | 0.038 | (-0.000, 0.076) * | 4.4 (73) |
| Coping | 0.029 | (-0.011, 0.069) | 4.0 (67) |
| Carer relationships | 0.011 | (-0.033, 0.054) | 4.1 (68) |
| Familial Relationships | **0.047** | **(0.015, 0.079)** *** | 6.2 (103) |
| Living Arrangements | **0.040** | **(0.004, 0.075)** ** | 5.1 (85) |
| Finances | 0.026 | (-0.006, 0.058) | 6.2 (103) |

Results are displayed in the format: coefficient (95% CI). Bolding denotes significance at the α = 0.05 level or less. Asterisks denote the following levels of significance: * significant at the α = 0.10 level, ** significant at the α = 0.05 level, and *** significant at the α = 0.01 level. Regressions were adjusted for all sociodemographic and clinical factors applied in univariable regressions.

**Supplementary Table 2** Verbatim quotations evidencing themes in qualitative data

| Quotations indicating isolation from friends and family |
| --- |
| A lack of personal contact with friends & family makes you feel very alone. A personal chat & laugh together is very important to help stop you feeling down, [and it] just isn't happening. (Female, moderate disability, exposed to the lockdown). |
| Supposed to have gone to the UK for [a] family wedding … So[sic] this [COVID-19] was very upsetting. Can't visit three of our sons and our grandchildren as they live outside of [our state]. (Female, no disability, unexposed to the lockdown). |
| I wonder when I will ever see them [family] again, particularly family in [the] UK. It is a horrible feeling but at least we have Facetime and WhatsApp to stay connected! (Female, no disability, unexposed to the lockdown). |
| Inability to visit children/grandchildren causes sadness, particularly in stage 4 lockdown. (Female, no disability, exposed to the lockdown). |
| My few ‘independent’ pleasures included meeting friends for coffee, which[sic] has been mostly forfeited. I miss physical contact ... especially HUGS! (Female, moderate disability, unexposed to the lockdown). |
| My main concern is that I am rarely interacting with my aged parents, other family, and friends. (Female, moderate disability, unexposed to the lockdown). |
| I feel like I've been lucky regarding the pandemic so far, with my main worries being for my children who live in Melbourne. I've also felt sad that we've been unable to visit for much longer than usual. And[sic] in general the social distancing and being unable to give friends and family a hug, and limited socialising, has been difficult. (Female, mild disability, unexposed to the lockdown). |
| My daughter lives in Melbourne and I feel very sorry that I can’t see her, nor can she visit me here … I think she is becoming depressed by the lockdowns that are happening. That is the reason I feel a bit emotional about the COVID-19 pandemic. (Female, no disability, unexposed to the lockdown). |
| I am living under stage three restrictions - dramatically restricting all contact face to face with family and friends, and excluding social activities with others. (Female, mild disability, exposed to the lockdown). |
| I have been working from home and feeling very alone. Being unable to see my new grandson has made me very miserable. (Female, moderate disability, exposed to the lockdown). |
| I have felt sometimes angry that this has restricted seeing my daughter … who lives in [a city], who is halfway through her pregnancy with my first grandchild, and I can only see her progress through photos or facetime[sic]. (Female, moderate disability, unexposed to the lockdown). |

| Quotations indicating a reduction in social and disease management activities |
| --- |
| Inability to continue voluntary community commitments has been one of the more difficult parts of Covid[sic] lockdown. Great emotional/social/intellectual satisfaction is gained by my volunteering, and this is the single most 'missed' part of my pre-COVID life. (Female, moderate disability, exposed to the lockdown). |
| Covid[sic] has stopped all of my social and health activities which help me emotionally as well as physically. This has been very difficult, and I am finding it hard to know what to do to find other activities that I can do that will help me. (Female, moderate disability, unexposed to the lockdown). |
| Physically my MS has worsened - no swimming in [the] pool, no Botox, [and] less physical therapy. (Female, severe disability, unexposed to the lockdown). |
| Lots of hands-on therapy/health services have been inaccessible for 6 months and it is taking a physical toll. (Female, mild disability, exposed to the lockdown). |
| It has been difficult to have my normal support & care on number of occasions due to risk of them coming to my home … Unable to do physical external exercises program, not allowed to go to therapeutic massage many times … Was unable to go to a specialist doctor in April, had to wait until August. Couldn't always go to appointments out of my area. Didn't get out and about for as much exercise. (Female, moderate disability, unexposed to the lockdown). |
| We are in stage 4 restrictions due to Covid 19 and have to wear a mask whenever we are outside. Stage 4 has had an impact on our daily activities, and we are unable to have any visitors or go further than 5 kms from our home. (Female, moderate disability, exposed to the lockdown). |
| Before COVID and the resulting gym closures I was a keen exerciser attending aqua aerobics and stretch *(and)* also Pilates classes and have not resumed any of those classes but have taken up walking instead. I miss organised classes as I lack motivation to exercise at home by myself. (Female, mild disability, unexposed to the lockdown). |
| My regular routine is no longer. I was unable to see my neurologist for regular appts. Unable to have myotherapy weekly [which] has impacted my mobility and increased my pain levels. (Female, moderate disability, exposed to the lockdown). |
| Medically[sic] delayed appointments and Ocrevus infusion due to pandemic numbers and hospital closures. In last month have been catching up on appointments. (Female, moderate disability, unexposed to the lockdown). |
| I am unable to access my NDIS capacity building activities - this is having an adverse effect on my mobility and emotional well-being. (Female, moderate disability, exposed to the lockdown). |

| Quotations indicating financial and emotional strain |
| --- |
| COVID 19[sic] has created an ever present[sic] fear and sadness for most people - having MS has increased my fear of vulnerability to more serious outcomes. Sometimes it feels like a dark cloud that is smothering me. (Female, moderate disability, unexposed to the lockdown). |
| Emotionally COVID-19 has definitely had an impact. The news alone has [had] a huge impact let alone worry about family members with health and financial worries, and friends who have MS. (Female, mild disability, unexposed to the lockdown). |
| Covid restrictions have stopped me seeing my family in [their state] and friends here, it's bad in Melbourne. I have suffered severe … depression as a result of being unwell (I have many other conditions which compounded my stress), and my business has to be transferred to online, but I spent 3 months extremely ill so now have lost 95% of my business. I am usually positive and calm but this time I feel lost. (Female, no disability, exposed to the lockdown). |
| I think the uncertainty of how long it will last and how bad it might get adds to the general sense of depression and anxiety. (Female, mild disability, unexposed to the lockdown). |
| I have been working from home since Stage 4 Restrictions were introduced in Victoria. I have felt extremely side-lined by the company I work for; not informed or invited to meetings … this has had the biggest impact on my self-worth, confidence, faith in my skills and abilities and led to sleeplessness, feeling down, depressed … I was told to work 2 x 1/2 days per week after working 4 very full days; however, my salary wasn't changed, but [I] live with concern that it will be reduced. (Female, no disability, exposed to the lockdown). |
| I have lost income from rental property and … my children have lost their jobs. This is very stressful especially when my physical ability to help in a practical way is gone. (Female, moderate disability, unexposed to the lockdown). |

| Quotations indicating benefit |
| --- |
| Staying at home (not working) has allowed me to focus more on my diet, with benefits to by[sic] weight, mood, and energy levels. (Female, no disability, unexposed to the lockdown). |
| Although being in stage 4 shut down, my physical and emotional health has been good. Thanks to modern technology we have family facetime, [and] keep in touch with friends. My husband and I love doing puzzles and word games. I am a quilter, love reading, knitter[sic] and making masks for my family, which fills up our days and keep our brains active. (Female, severe disability, exposed to the lockdown) |
| While I do miss my face-to-face volunteer role and actual rather than virtual coffee catch ups with friends, there have been some silver linings. I do freelance editing from home and the available work has increased if anything. Also, as I am a wheelchair user, the pandemic-driven increase in online events has significantly increased the number of entertainment, cultural and educational events I can attend. I hope that online offerings will continue to some extent post-lockdown. (Female, severe disability, exposed to the lockdown). |
| It’s[sic] been wonderful to have my husband home with me 7 days per week! (Male, severe disability, unexposed to the lockdown). |
| In reference to financial impact only, I am better off on Jobseeker. This stems from absences and health care costs. I have known for some time that my hearing has been failing and now with the pensioner concessions I have bought a hearing aid. Ability to recall from super has also allowed me to reduce credit card and other debts. (Male, mild disability, unexposed to the lockdown). |
| Because of not travelling or visiting I have been quietly[sic] at home which has been relaxing. No expectations to visit children etc. Spent time using [a] treadmill to lose weight to help overall health. (Female, mild disability, exposed to the lockdown). |
| The biggest impact is increased opportunity and ability to work effectively from home rather than in the office, a positive one for me. (Male, moderate disability severity, exposed to the lockdown). |
| I have been given the opportunity to work from home which has had a massive influence on my fatigue levels, and also my anxiety levels. (Female, moderate disability, unexposed to the lockdown). |

**Supplementary Table 3** Further results from ordered probit regressions on COVID-19-related adversity variables

|  | Self-care activities | | Coping with MS | | Self-care activities | |
| --- | --- | --- | --- | --- | --- | --- |
| Age (years) | **-0.027** | **(-0.037, -0.017)** *** | **-0.019** | **(-0.027, 0.011)** *** | **-0.033** | **(-0.059, -0.008)** *** |
| Sex (female) | -0.077 | (-0.279, 0.125) | -0.090 | (-0.258, 0.078) | -0.080 | (-0.380, 0.219) |
| Employment (employed) | **-0.295** | **(-0.516, -0.074)** *** | -0.142 | (-0.314, 0.031) | **-0.370** | **(-0.728, -0.013)** ** |
| Phenotype (relapse-onset) | 0.155 | (-0.124, 0.433) | **0.267** | **(0.028, 0.505)** ** | **0.523** | **(0.069, 0.976)** ** |
| Education |  |  |  |  |  |  |
| (1) Occupational | -0.042 | (-0.255, 0.170) | -0.057 | (-0.239, 0.126) | -0.053 | (-0.267, 0.372) |
| (2) Bachelor’s degree | -0.228 | (-0.472, 0.017) * | -0.160 | (-0.371, 0.051) | -0.129 | (-0.506, 0.248) |
| (3) Postgraduate degree | -0.032 | (-0.289, 0.225) | -0.013 | (-0.231, 0.205) | -0.027 | (-0.716, 0.167) |
| Disability severity |  |  |  |  |  |  |
| (1) Mild | -0.002 | (-0.383, 0.379) | **0.266** | **(0.075, 0.457)** *** | **0.508** | **(0.120, 0.896)** *** |
| (2) Moderate | **0.417** | **(0.155, 0.678)** *** | **0.431** | **(0.244, 0.619)** *** | **1.205** | **(0.594, 1.817)** *** |
| (3) Severe | 0.304 | (-0.062, 0.670) | 0.209 | (-0.074, 0.493) | **1.023** | **(0.477, 1.569)** *** |
| Socioeconomic area |  |  |  |  |  |  |
| (1) Quartile two | -0.012 | (-0.336, 0.312) | 0.075 | (-0.183, 0.333) | -0.074 | (-0.521, 0.372) |
| (2) Quartile three | -0.287 | (-0.606, 0.032) * | -0.069 | (-0.331, 0.192) | -0.327 | (-0.793, 0.139) |
| (3) Quartile four | -0.004 | (-0.311, 0.303) | 0.162 | (-0.092, 0.416) | 0.024 | (-0.413, 0.462) |
| (4) Quartile five | 0.031 | (-0.261, 0.324) | 0.120 | (-0.128, 0.369) | -0.133 | (-0.551, 0.285) |
| Lockdown |  |  |  |  |  |  |
| (1) Metropolitan | **0.597** | **(0.383, 0.812)** *** | **0.562** | **(0.367, 0.756)** *** | **1.114** | **(0.532, 1.697)** *** |
| (2) Regional | **0.651** | **(0.234, 1.068)** *** | **0.429** | **(0.119, 0.739)** *** | **0.994** | **(0.320, 1.669)** *** |
| Cut one | -0.672 | (-1.415, 0.071) | -0.427 | (-1.048, 0.195) | -0.161 | (-1.283, 0.961) |
| Cut two | 0.219 | (-0.488, 0.927) | 0.551 | (-0.052, 1.154) | 1.266 | (-0.204, 2.328) |
|  | Familial relationships | | Living Arrangements | | Finances | |
| Age (years) | -0.004 | (-0.012, 0.003) | -0.006 | (-0.012, 0.001) | **-0.009** | **(-0.016, 0.002)** *** |
| Sex (female) | 0.017 | (-0.162, 0.196) | -0.086 | (-0.232, 0.061) | -0.091 | (-0.251, 0.070) |
| Employment (employed) | **-0.213** | **(-0.396, -0.029)** ** | -0.099 | (-0.269, 0.071) | -0.050 | (-0.240, 0.140) |
| Phenotype (relapse-onset) | **0.285** | **(0.033, 0.536)** ** | 0.059 | (0.129, 0.247) | **0.245** | **(0.027, 0.463)** ** |
| Education |  |  |  |  |  |  |
| (1) Occupational | 0.081 | (-0.106, 0.267) | -0.006 | (-0.165, 0.153) | -0.020 | (-0.195, 0.155) |
| (2) Bachelor’s degree | -0.164 | (-0.378, 0.050) | -0.135 | (-0.322, 0.052) | -0.027 | (-0.229, 0.175) |
| (3) Postgraduate degree | -0.111 | (-0.343, 0.122) | 0.067 | (-0.129, 0.263) | -0.026 | (-0.250, 0.197) |
| Disability severity |  |  |  |  |  |  |
| (1) Mild | 0.094 | (-0.107, 0.294) | **0.262** | **(0.075, 0.449)** *** | 0.039 | (-0.162, 0.240) |
| (2) Moderate | **0.251** | **(0.045, 0.457)** ** | **0.425** | **(0.250, 0.599)** *** | **0.264** | **(0.077, 0.450)** *** |
| (3) Severe | 0.181 | (-0.075, 0.436) | **0.428** | **(0.218, 0.639)** *** | 0.081 | (-0.160, 0.322) |
| Socioeconomic area |  |  |  |  |  |  |
| (1) Quartile two | 0.014 | (-0.260, 0.287) | -0.100 | (-0.327, 0.126) | -0.050 | (-0.184, 0.293) |
| (2) Quartile three | -0.058 | (-0.329, 0.214) | -0.122 | (-0.354, 0.314) | -0.084 | (-0.154, 0.347) |
| (3) Quartile four | 0.066 | (-0.197, 0.329) | 0.089 | (-0.136, 0.109) | 0.097 | (-0.341, 0.174) |
| (4) Quartile five | 0.074 | (-0.180, 0.327) | 0.007 | (-0.208, 0.223) | 0.054 | (-0.184, 0.293) |
| Lockdown |  |  |  |  |  |  |
| (1) Metropolitan | **0.650** | **(0.446, 0.854)** *** | **0.369** | **(0.223, 0.514)** *** | **0.253** | **(0.093, 0.413)** *** |
| (2) Regional | **0.775** | **(0.423, 1.128)** *** | 0.101 | (-0.174, 0.378) | 0.124 | (-0.180, 0.428) |
| Cut one | 0.346 | (-0.279, 0.971) | 0.311 | (-0.258, 0.880) | 0.513 | (-0.089, 1.114) |
| Cut two | 1.121 | (0.500, 1.741) | 1.010 | (0.440, 1.580) | 1.006 | (0.402, 1.611) |

Notes: Results are displayed in the format: coefficient (95% CI). Bolding denotes significance at the α = 0.05 level or less. Asterisks denote the following levels of significance: * significant at the α = 0.05 level, ** significant at the α = 0.05 level, and *** significant at the α = 0.01 level. Measures of significance do not apply to cut points. Coefficients in the table represent sentiment associated with higher adversity rankings, and the cut-points represent levels of sentiment which must be reached before a participant is likely to report a higher level of adversity. If cut one is exceeded a participant is most likely to report minor adversity. Similarly, if cut two is exceeded a participant is most likely to report major adversity.

**Supplementary Table 4** Sensitivity analysis for the lockdown variable, regional category, using composite adversity as an outcome measure

|  | ^d^ More inclusive | | ^e^ Less inclusive | | ^f^ Original | |
| --- | --- | --- | --- | --- | --- | --- |
| Age (years) | **-0.013** | **(-0.018, -0.007)** *** | **-0.013** | **(-0.018, -0.007)** *** | **-0.013** | **(-0.018, -0.008)** *** |
| Sex (female) | 0.021 | (-0.097, 0.139) | -0.008 | (-0.112, 0.129) | 0.020 | (-0.098, 0.139) |
| Employment (employed) | -0.115 | (-0.259, 0.029) | -0.113 | (-0.259, 0.032) | -0.126 | (-0.256, 0.005) * |
| Phenotype (relapse-onset) | **0.180** | **(0.026, 0.333)** ** | **0.287** | **(0.030, 0.344)** ** | **0.165** | **(0.011, 0.319)** ** |
| Education |  |  |  |  |  |  |
| (1) Certificate or diploma | 0.059 | (-0.091, 0.210) | 0.067 | (-0.088, 0.221) | -0.050 | (-0.102, 0.202) |
| (2) Bachelor’s degree | -0.051 | (-0.230, 0.128) | -0.046 | (-0.230, 0.138) | -0.062 | (-0.244, 0.120) |
| (3) Postgraduate degree | 0.051 | (-0.127, 0.228) | 0.055 | (-0.128, 0.239) | 0.038 | (-0.144, 0.220) |
| Disability severity |  |  |  |  |  |  |
| (1) Mild | **0.176** | **(0.033, 0.319)** ** | **0.175** | **(0.031, 0.320)** *** | **0.177** | **(0.035, 0.320)** ** |
| (2) Moderate | **0.431** | **(0.277, 0.585)** *** | **0.443** | **(0.287, 0.600)** *** | **0.425** | **(0.272, 0.577)** *** |
| (3) Severe | **0.351** | **(0.171, 0.531)** *** | **0.356** | **(0.173, 0.539)** *** | **0.337** | **(0.159, 0.516)** *** |
| Socioeconomic area |  |  |  |  |  |  |
| (1) Quintile two | 0.050 | (-0.143, 0.244) | 0.027 | (-0.171, 0.224) | 0.049 | (-0.147, 0.245) |
| (2) Quintile three | -0.067 | (-0.253, 0.118) | -0.094 | (-0.282, 0.093) | -0.073 | (-0.259, 0.114) |
| (3) Quintile four | 0.151 | (-0.036, 0.337) | 0.129 | (-0.060, 0.318) | 0.151 | (-0.038, 0.339) |
| (4) Quintile five | 0.142 | (-0.039, 0.322) | 0.105 | (-0.076, 0.284) | 0.138 | (-0.048, 0.323) |
| Lockdown |  |  |  |  |  |  |
| (1) Metropolitan | **0.488** | **(0.363, 0.614)** *** | **0.476** | **(0.350, 0.601)** *** | **0.479** | **(0.354, 0.603)** *** |
| (2) Regional | **0.476** | **(0.280, 0.672)** *** | **0.426** | **(0.135, 0.718)** *** | **0.435** | **(0.225, 0.645)** *** |
| *Cut one* | 0.011 | (-0.425, 0.446) | -0.024 | (-0.466, 0.419) | -0.044 | (-0.470, 0.382) |
| *Cut two* | 1.046 | (0.600, 1.492) | 1.025 | (0.578, 1.473) | 0.990 | (0.557, 1.423) |

^d^ $n=86$ participants in $LOCK_{i}$ category 2. Applies to all participants exposed to the regional lockdown, regardless of the duration of exposure.

^e^ $n=44$ participants in $LOCK_{i}$ category 2. Applies to participants exposed to the regional lockdown for at least two weeks.

^f^ $n=68$ participants in $LOCK_{i}$ category 2. Applies to participants exposed to the regional lockdown for at least one week.

Results are displayed in the format: coefficient (95% CI). Bolding denotes significance at the α = 0.05 level or less. Asterisks denote the following levels of significance: * significant at the α = 0.10 level, ** significant at the α = 0.05 level, and *** significant at the α = 0.01 level. Measures of significance do not apply to cut points.

The above table displays the results of a sensitivity analysis, undertaken to determine whether different definitions of exposure to the regional lockdown would cause significant differences in coefficient estimates. While results in the table were obtained using composite adversity as the dependent variable, similar analyses were also undertaken which utilised the other COVID-19-related adversity variables. The sensitivity analyses did not show coefficient estimates to differ significantly with regional lockdown exposure definitions, with dissimilarities explicable by random chance.

**References**

1. Cranmer, S. J., & Gill, J. (2013). We Have to Be Discrete About This: A Non-Parametric Imputation Technique for Missing Categorical Data. *Br J Political Sci*, 43(2), 425-449. https://doi.org/10.1017/S0007123412000312

2. Kropko, J., Goodrich, B., Gelman, A., & Hill, J. (2014). Multiple imputation for continuous and categorical data: comparing joint multivariate normal and conditional approaches. *Political Anal*, 22(4). https://doi.org/10.1093/pan/mpu007

3. Azur, M. J., Stuart, E. A., Frangakis, C., & Leaf, P. J. (2011). Multiple imputation by chained equations: what is it and how does it work? *Int J Methods Psychiatr Res*, 20(1), 40-49. https://doi.org/10.1002/mpr.329

4. Wooldridge, J. M. (2010). *Econometric analysis of cross section and panel data*. MIT Press.

5. Harvey, A. C. (1976). Estimating Regression-Models with Multiplicative Heteroscedasticity. *Econometrica*, 44(3), 461-465. https://doi.org/10.2307/1913974

6. Hill, R. C., Griffiths, W. E., & Lim, G. C. (2018). *Principles of Econometrics*. John Wiley & Sons.
